# Practical Notes and Formulæ

**Published:** 1883-03-20

**Authors:** 


### PRACTICAL NOTES AND FORMULÆ.

**Antiseptic Cologne.**—The following is commended as a preparation combining antiseptic properties with a perfume—

| | |
|---|---|
| Eau de cologne | 8 fl. ounces, |
| Chloral hydrate | 2 drachms, |
| Quinine (alkaloid) | 10 grains, |
| Carbolic acid (pure) | 30 grains, |
| Oil of lavender | 20 drops. |

The Medical Record says this may be used on the handkerchief, the doctor holding it gently to the mouth while in the sick-room. Warranted to keep out bacillus tuberculosis; also, b. termo, b. elephantiasis A., and b. micrococci.—*Drug. Circular.*

**Cough Medicines.**—We present a few simple recipes for expectorants, useful for winter coughs. The first is particularly useful for young children—

| | |
|---|---|
| Syrup of squill | 1 fl. drachm, |
| Gum acacia, powdered | ½ fl. drachm, |
| Ammonium chloride | 8 grains. |

Peppermint water, enough to make two fluid ounces.

Dose for a child, a teaspoonful every two hours.

Another formula for adults and older children, consists of—

| | |
|---|---|
| Syrup of ipecac | 2 parts, |
| Syrup of squill | 4 parts, |
| Paregoric | 1 part. |

Dose, half to one teaspoonful, repeated as often as necessary.

The following was a favorite prescription of the late Professor C. A. Lee, of Peekskill—

| | |
|---|---|
| Syrup of ipecac | 1 ounce, |
| Syrup of tolu | 1 " |
| Paregoric | ½ " |
| Syrup of wild cherry | 1 " |

—*Drug. Circular.*

**A Subscriber** to the Medical Call writes that the mother tincture of *gelsemium*, given in drop doses every ten, twenty or thirty minutes, relieves ninety-nine per cent. of her cases of dysmenorrhœa. If taken three or four times a day for about three days before the period, no severe pain will be experienced —*N. Y. Times.*

**The Metric System**—To convert grains or minims into centigrammes, multiply them by six; to convert drachms into grammes, multiply them by four; to convert ounces into grammes, multiply them by thirty-two.—*Ex.*

**Diarrhœa Pills.**—The following formula is given by Professor Wm. Thompson, of the University of the city of New York—

| | | |
|---|---|---|
| ℞ | Plumbi acetatis | grs. 16, |
| | Pulv. camphoræ | grs. 12, |
| | Pulv. opii | grs. 3, |
| | Bismuth subcarb | grs. 12, |
| | Extract gentian | q. s. |

M. Make a mass and divide into 12 pills.—*Drug. Circular.*

**Comparative Temperatures.**—In a series of observations by Dr. Taylor (Roosevelt Hospital Medical Record), it is stated—

*First.*—The difference between axillary and rectal and axillary and vaginal temperatures is not constant, but averages about 1° F. in favor of the rectum and vagina.

*Second.*—In certain exceptional cases the temperature may be considerably higher in the axilla than in the rectum or vagina.

*Third.*—The difference does not seem to vary directly with the height of the temperature.

*Fourth.*—The difference in favor of the mouth, in buccal and axillary temperatures, averages about one-half that in favor of the rectum and vagina, when axillary temperatures are accompanied with the latter.

**Toothache Drops.**—

| | | |
|---|---|---|
| R | Spr. chloroform | aa ℥ fld. M. |
| | Spr. ether sulph | |
| | Spr. camphor | |
| | Tr. opii | |
| | Tr. iodini | |

**Facts Worth Knowing.**—That salt fish are quickest and best freshened by soaking in sour milk.

That cold rain water and soap will remove machine grease from washable fabrics.

That fish may be scaled much easier by first dipping them into boiling water for a minute.

That fresh meat, beginning to sour, will sweeten if placed out of doors in the cool air over night.

That milk which has changed may be sweetened or rendered fit for use again by stirring in a little soda.

That boiling starch is much improved by the addition of sperm, or salt, or both, or a little gum arabic, dissolved.

That a tablespoonful of turpentine, boiled with your white clothes, will greatly aid the whitening process.

That kerosene will soften boots and shoes that have been hardened by water, and will render them pliable as new.

That clear boiling water will remove tea stains; pour the water through the stain, and thus prevent its spreading over the fabric. —*Journal of Health.*

